# Hyaluronic acid selective anchoring to the cytoskeleton: An atomic force microscopy study

**DOI:** 10.1371/journal.pone.0206056

**Published:** 2018-10-25

**Authors:** Stefania Marcotti, Koichiro Maki, Gwendolen C. Reilly, Damien Lacroix, Taiji Adachi

**Affiliations:** 1 Insigneo Institute for in silico Medicine, University of Sheffield, Sheffield, United Kingdom; 2 Department of Mechanical Engineering, University of Sheffield, Sheffield, United Kingdom; 3 Randall Centre for Cell and Molecular Biophysics, King’s College London, London, United Kingdom; 4 Department of Biosystems Science, Institute for Frontier Life and Medical Sciences, Kyoto University, Kyoto, Japan; 5 Department of Mechanical Engineering, University of Tokyo, Tokyo, Japan; 6 Department of Materials Science and Engineering, University of Sheffield, Sheffield, United Kingdom; University of California Davis, UNITED STATES

## Abstract

The hyaluronic acid component of the glycocalyx plays a role in cell mechanotransduction by selectively transmitting mechanical signals to the cell cytoskeleton or to the cell membrane. The aim of this study was to evaluate the mechanical link between the hyaluronic acid molecule and the cell cytoskeleton by means of atomic force microscopy single molecule force spectroscopy. Hyaluronic acid molecules on live cells were targeted with probes coated with hyaluronic acid binding protein. Two different types of events were observed when the detachment of the target molecule from the probe occurred, suggesting the presence of cytoskeleton- and membrane-anchored molecules. Membrane-anchored molecules facilitated the formation of tethers when pulled. About 15% of the tested hyaluronic acid molecules were shown to be anchored to the cytoskeleton. When multiple molecules bonded to the probe, specific detachment patterns were observed, suggesting that a cytoskeletal bond needed to be broken to improve the ability to pull tethers from the cell membrane. This likely resulted in the formation of tethering structures maintaining a cytoskeletal core similar to the ones observed for cells over-expressing HA synthases. The different observed rupture events were associated with separate mechanotransductive mechanisms in an analogous manner to that previously proposed for the endothelial glycocalyx. Single cytoskeleton anchored rupture events represent HA molecules linked to the cytoskeleton and therefore transmitting mechanical stimuli into the inner cell compartments. Single membrane tethers would conversely represent the glycocalyx molecules connected to areas of the membrane where an abundance of signalling molecules reside.

## Introduction

Hyaluronic Acid (HA) is a glycosaminoglycan composed of repeated disaccharide units in the form of a linear polymer [1]. It is synthesised by three related trans-membrane proteins (HAS1, HAS2, HAS3), extruded towards the outer surface of cells [2] and cleaved by specific enzymes (hyaluronidases, HAase) [3]. HA is involved in various physiological cell functions and is considered to be a contributor to mechanotransduction and signal mediation [2]. Its mechanical and swelling properties can tune cellular functions such as adhesion and spreading and it can form structures, such as cables [4] and microvilli [5–7], which can play a role in signal transmission. Furthermore, HA has the ability to change local membrane properties acting as an external cytoskeleton by modifying and controlling the cell shape [8].

In conjunction with proteoglycans and other non-proteoglycan components, HA forms the cell glycocalyx, a membrane-bound collection of macromolecules on the outer surface of cells belonging to different tissues [9–13] which has been investigated as a cell mechanotransducer [14–17]. Different hypotheses have been formulated to explain the underlying mechanisms of glycocalyx-mediated mechanotransduction [16,18,19]. Firstly, a “decentralised” mechanism could take place, where the mechanosensing happens at the glycocalyx level while the mechanotransduction at sites distinct from the surface (i.e. cytoskeleton, focal adhesions and nucleus). The glycocalyx fibre deflection due to fluid shear stress would cause molecular displacement of signalling proteins on the cell cytoskeleton [20]. In addition to this “decentralised” mechanism, a “centralised” mechanism could also occur for which the glycocalyx acts as a mechanosensor and a mechanotransducer. This would be mediated by glycocalyx fibres directly connected to the membrane *caveolae* where an abundance of signalling molecules reside [16].

The connection between the glycocalyx/HA and the cell cytoskeleton appears to be crucial for signal mediation and for exploring the occurrence of the different hypothesised mechanotransduction mechanisms. HA is anchored to the cell through its synthases or through surface receptors, such as CD44 [2]. It has been hypothesised that both synthases [5] and CD44 [21] could selectively bind to the actin cytoskeleton and the actin-binding link molecules have been identified for the CD44 receptor in the ERM (ezrin-radixin-moesin) protein family and in the related protein merlin. CD44 has no actin-binding sites on its cytoplasmic domain, suggesting an indirect interaction mediated by these cytoskeleton-associated proteins. Both these link molecules have active and inactive forms allowing switch-like binding between HA and the actin cytoskeleton [21]. Mechanotransductive roles were hypothesised for ezrin [22] and merlin [23], suggesting that these proteins are good candidates for mechanical signal transmission from the outer to the inner cell compartments through the glycocalyx.

Recently, an Atomic Force Microscopy (AFM) single-molecule force spectroscopy methodology was developed to evaluate the mechanical attachment of a target molecule to the cytoskeleton in case of switch-like anchoring mechanisms [24–27]. This was achieved by analysing the force-distance curve in the proximity of the rupture events between the probe and the target molecule. In the present study, a similar methodology was employed to investigate the HA connection to the cytoskeleton of live cells. Murine pre-osteoblast MC3T3-E1 cells were used, which are known to have an HA-rich glycocalyx involved in mechanotransduction [28] and to express CD44 under similar culture conditions [29,30]. The rationale of the present work was to study the HA mechanical linkage to the actin cytoskeleton by means of AFM single-molecule force spectroscopy of HA on the surface of bone cells. The occurrence of rupture events in the case of a normal or degraded glycocalyx could give an insight into the possible mechanisms of signal transduction between the outer and the inner compartments of cells. Moreover, a quantitative measure of the HA molecules bound or not bound to the actin cytoskeleton could be calculated.

## Materials and methods

### 1. Cells

Murine pre-osteoblast cells MC3T3-E1 (Riken cell bank, passage 22–23, [31]) were cultured in Minimum Essential Alpha Eagle medium (Lonza) supplemented with nucleosides and 2 mM UltraGlutamine I, 10% v/v foetal bovine serum (Labtech) and a solution of 100 units/ml penicillin and 100 μg/ml streptomycin (Sigma-Aldrich). Cells were kept at 37°C and passaged when 70% confluent. Prior to AFM analysis, cells were seeded on tissue culture plastic dishes (D = 36 mm, seeding density 1200 cell/cm^2^) and tested after 4 to 5 days of culture when reaching 45%-85% confluence. If not confluent, MC3T3 cells are not expected to produce mineral and were, therefore, considered to be in the pre-osteoblastic differentiation stage [31]. MC3T3 cells are known to have an HA-rich glycocalyx involved in mechanotransduction [28] and to express CD44 [29,30] under similar culture conditions. To the best of our knowledge, no information is available on the predominant CD44 isoforms or on the expression of the HA synthases in these specific cells, which is, however, expected to be similar to other cell types displaying an HA-rich glycocalyx [5–7].

### 2. Samples

Hyaluronic Acid Binding Protein (HABP, from bovine nasal cartilage, Merck) was used to specifically bind HA on the cell surface. This molecule is commonly employed for the detection of HA in cell culture [32] and it is composed of the HA binding domain of aggrecan and its native stabiliser link molecule [33–35].

Four different samples were employed for experiments, designed as follows:

1. HABP/HA: cantilever functionalised with HABP, untreated cell sample;
2. BSA/HA: cantilever functionalised with bovine serum albumin (BSA, Sigma-Aldrich), untreated cell sample;
3. untreated/HA: non-functionalised cantilever, untreated cell sample;
4. HABP/HAase: cantilever functionalised with HABP, cell sample treated with HAase.

Sample 1 (HABP/HA) represented the study experiment, with the cantilever functionalised to target the HA molecules on cells. Samples 2–4 were considered as controls, to verify the specificity of the protocol. In these cases, no pulling events should be observed. The BSA used for cantilever functionalisation in Sample 2 (BSA/HA) should block all the non-specific bonds. As a further verification, Sample 3 (untreated/HA) was designed with no cantilever functionalisation. In the case of Sample 4 (HABP/HAase), the HA was removed from the cell by enzymatic treatment with HAase (from *Streptomyces hyalurolyticus*, Sigma-Aldrich). To this aim, a previously described protocol [28] was used and cells were treated with 160 U/ml HAase/media for 1 hour prior to AFM experiments.

### 3. AFM

#### 3a. Cantilever functionalisation

Low spring constant cantilevers with pyramidal tip (Olympus) were used for all the experiments (nominal spring constant 0.02 N/m, tip radius 15 nm). A similar cantilever functionalisation method was described elsewhere [36,37]. The steps of activation are listed below and were the same for Sample 1 (HABP/HA, functionalisation molecule: HABP), Sample 2 (BSA/HA, functionalisation molecule: BSA) and Sample 4 (HABP/HAase, functionalisation molecule: HABP). The cantilevers used to test cells in Sample 3 (untreated/HA) were not treated. Detailed description of the cantilever functionalisation protocol can be found at the following DOI: http://dx.doi.org/10.17504/protocols.io.ra4d2gw.

The following activation steps were performed just before the experiments:

1. cantilevers were oxidised using an ozone cleaner and submerged in 2% w/w (3-Aminopropyl) triethoxysilane (APTES, Sigma-Aldrich)/ultra-pure water for 15 minutes to depose (-SH) groups on the probe surface;
2. after washing, the cantilevers were submerged in 6 mM Maleimide-C_3_-NTA (DOJINDO Laboratories)/Tris-HCl buffer for 30 minutes to expose NHS esters;
3. after washing, the functionalisation molecule was bound to the exposed NHS ester groups by submerging the cantilever in 100 nM HABP/Tris-HCl buffer solution (Sample 1 HABP/HA and Sample 4 HABP/HAase) or 1% w/v BSA/ultra-pure water (Sample 2 BSA/HA) for 1 hour;
4. the excess maleimide was quenched with 50 mM 2-mercaptoethanol (Sigma-Aldrich)/ultra-pure water by submerging the cantilevers for 1 minute;
5. after a final wash, the functionalised cantilevers were kept submerged in ultra-pure water until mounting on the AFM holder.

#### 3b. AFM set-up

A NanoWizard 3 Atomic Force Microscope (JPK) coupled to an IX series optical inverted microscope (Olympus) enclosed in a metal box to reduce environmental noise was used for all the experiments.

The cell sample was washed with phosphate buffer solution and fresh medium was added before testing for a maximum of 2 hours at room temperature. Cells were located through the optical microscope and tested within an area of 10 x 10 μm^2^. A 16-point grid was drawn and force spectroscopy measurements were obtained on the grid for three times to collect 48 data on each cell. The relative set point and the approach velocity were set to 0.5 nN and 2 μm/s respectively. A total of 150 cells were tested over 4 separate experiments for Sample 1 (HABP/HA); 15 cells were tested for each of the control samples.

### 4. Post-processing

The post-processing was performed in MATLAB (MathWorks) with custom-written algorithms. An automated algorithm was designed to fit the contact point on each extend curve with the ratio of variance method [38]. If an offset of the retract baseline was observed in respect to the extend baseline, this was considered to be due to the hydrodynamic effect of the cantilever movement in liquid and was corrected before further analysis by translating the retract curve so that the baselines would match [39]. All pre-processed data are available at the following DOI: 10.15131/shef.data.5632783.

Force-distance retract curves were analysed as the focus was on the pulling of the target molecules that occurred when the probe was moved away from the cell surface. A clear decrease in force representing the unbinding between the probe and the target molecule was defined as a rupture event. Multiple rupture events could be observed in one retract curve if multiple target molecules were initially bound to the probe. The force-distance ratio (measured in pN/nm) preceding the rupture event was described as “slope” and used to classify the rupture events.

#### 4a. Localisation of rupture events

Rupture events were searched for in the region of the force-distance curve where the probe was not in contact with the cell body, i.e. after the contact point. If a curve displayed any rupture events occurring in the indentation region (i.e. before the contact point), it was excluded from further analysis as this could be due to membrane piercing, an incomplete detachment of tethers in previous locations or noise.

**Fig 1:**
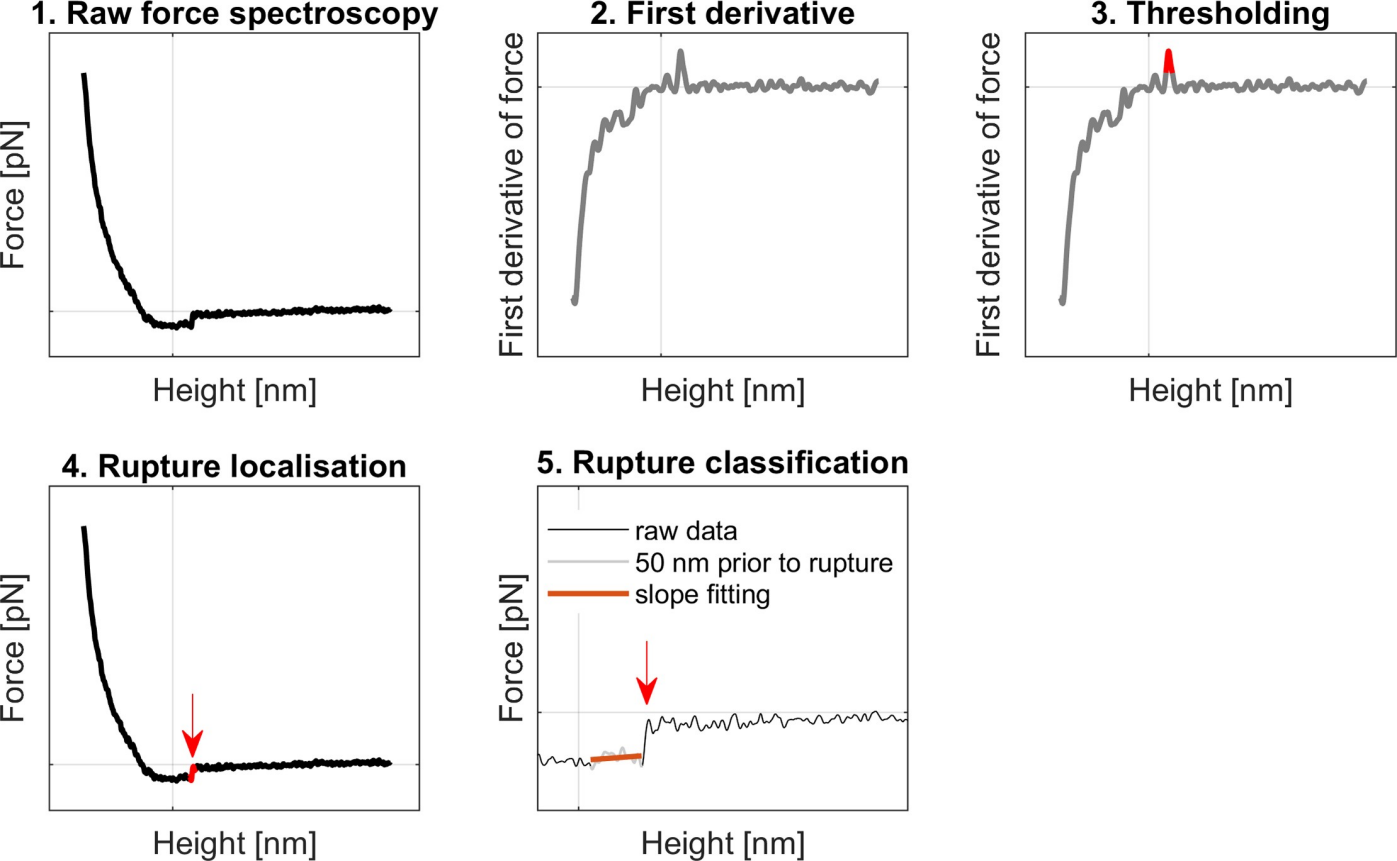
Protocol summary for rupture event localisation and classification. Each force spectroscopy curve (1) was analysed separately. After contact point fitting, the first derivative of the force was calculated (2). A threshold was set to locate peaks in the derivative (3) correspondent to rupture events (4). A data interval of 50 nm prior to the rupture event was fitted with a line (5); the slope was evaluated to classify the rupture event as cytoskeleton anchored or membrane tether.

The first derivative of the signal was screened to detect the rupture events after smoothing to remove high-frequency noise [26] (Fig 1). When a rupture event is encountered, the first derivative shows a peak due to the close-to-vertical line that marks the unbinding between the probe and the target molecule. These peaks were detected and their force and distance from the contact point were recorded. The slope in an interval of 50 nm preceding the rupture events was also computed.

#### 4b. Successful target rate

If a force-distance curve showed at least one rupture event it was considered as successfully targeting the molecule of interest (HA) on the cell surface. The number of successful target curves was counted over the total number of data curves for each sample as an estimate of the ease of binding between the probe and the target molecule for each pair.

#### 4c. Classification of rupture events

**Fig 2:**
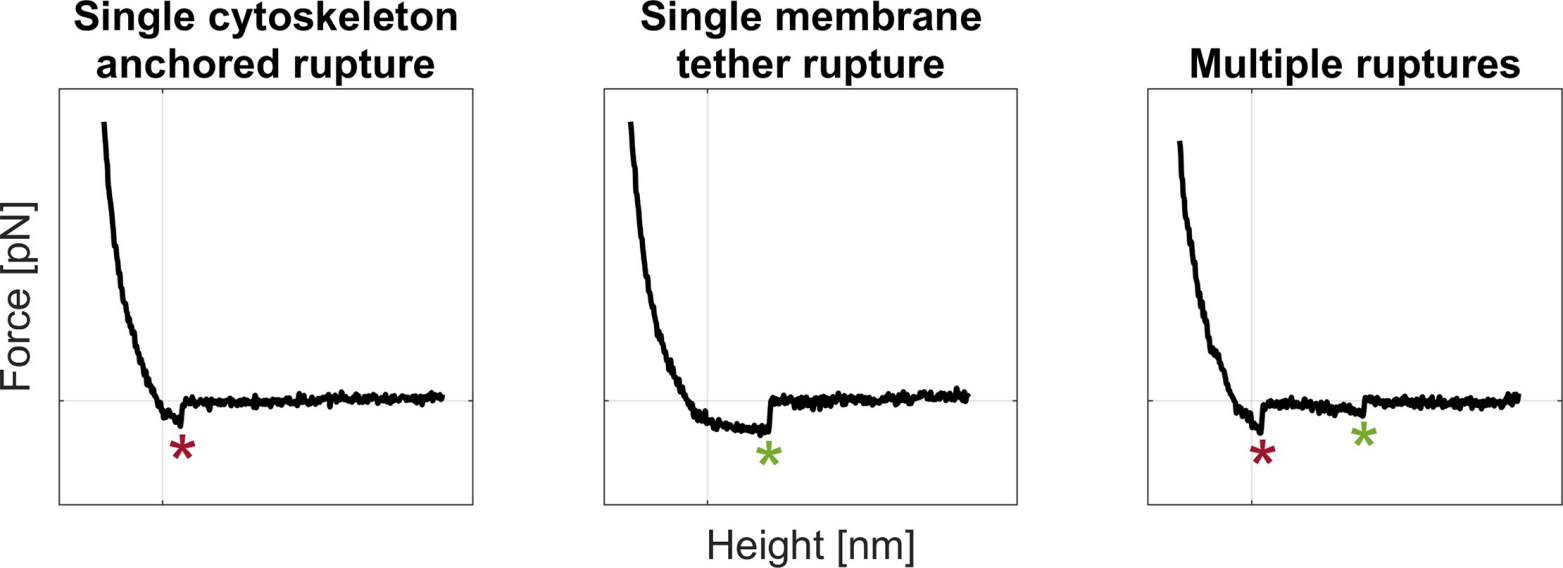
Example of rupture events. From left to right, representative AFM raw force spectroscopy data are shown for single cytoskeleton anchored ruptures (red asterisks), single membrane tether ruptures (green asterisks) and multiple ruptures, respectively.

The parameter defined as slope could act as a classifier for the rupture events. If the slope preceding the event was higher than a threshold, this would represent a cytoskeleton anchored rupture as a clear rise in force occurred. Otherwise, the event would be classified as a membrane tether rupture (i.e. non-anchored rupture), if preceded by long force plateau [25,26] (Fig 2). To define this threshold, it was necessary to evaluate the variability range of a force that could be described as constant, such as the baseline slope of the extend curves.

**Fig 3:**
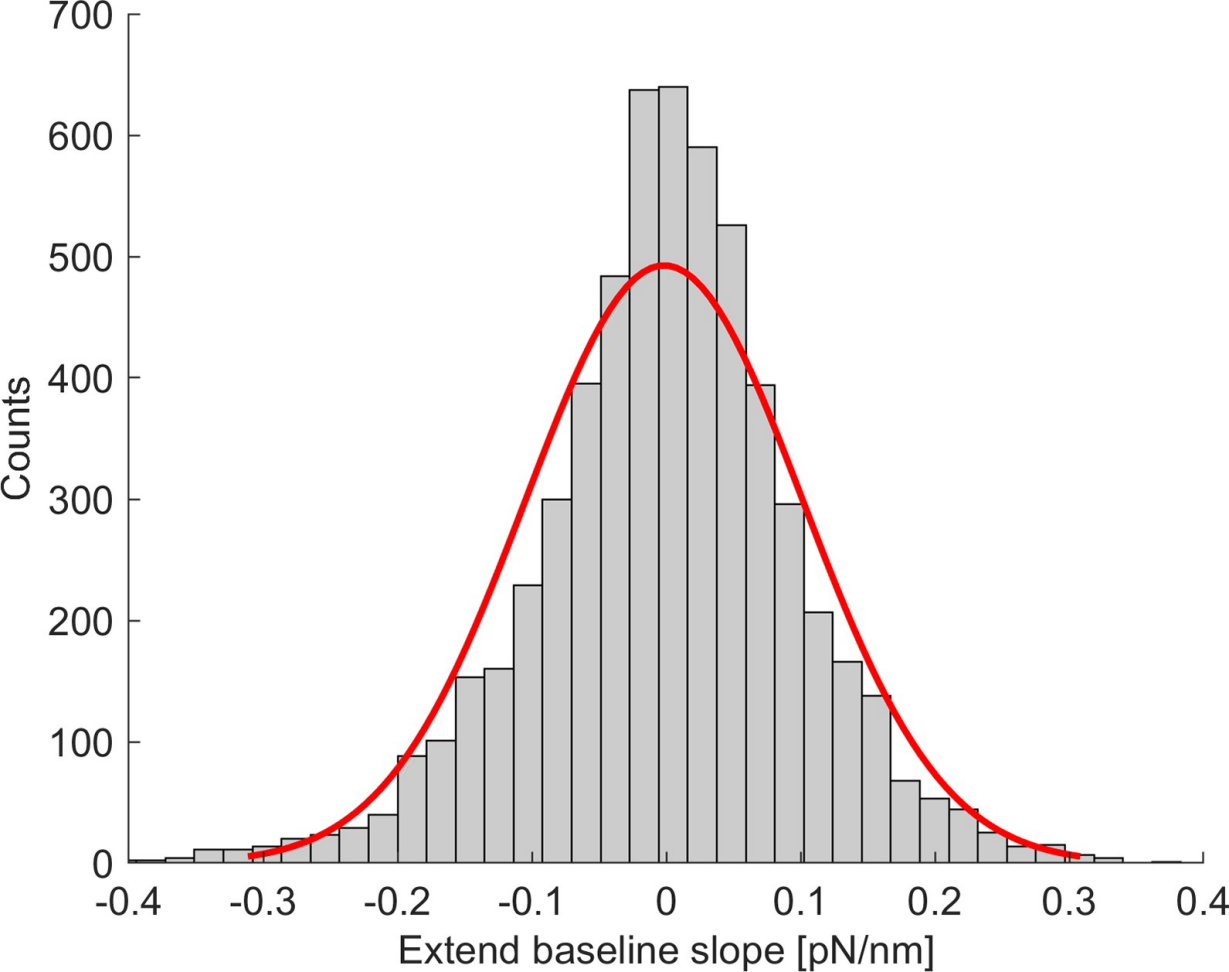
Histogram of the extend baseline slope to define the rupture classification threshold. The histogram of the slope of all extend baseline data is shown. The red line represents a normal distribution fit (μ = -0.002 pN/nm and σ = 0.103 pN/nm).

The slope of the last 50 nm of all extend baseline data was calculated (Fig 3). The resulting histogram was fitted with a normal distribution with the following descriptive parameters: μ = - 0.002 pN/nm and σ = 0.103 pN/nm. About 99.7% of data lies in the space defined by μ ± 3σ and therefore in the interval (-0.311 pN/nm, 0.307 pN/nm). This interval was used to define the membrane tether rupture slope variability range.

The membrane tether ruptures were classified with their associated force, calculated as the sudden release of force due to tether rupture. If multiple tethers were formed, the tether force corresponded to the force step between one tether and the subsequent one [40]. The tether length was computed as the distance of the rupture event from the contact point.

#### 4d. Single and multiple rupture events

A rupture event was classified as single if it was the only one in a given curve, as multiple otherwise (Fig 2). The percentage of single and multiple rupture events and their associated force were calculated to verify the occurrence of specific rupture patterns.

#### 4e. Intrinsic bond characterisation

In the present study, the experiments were carried out with a single loading rate and therefore the Bell-Evans model [41–43] could not be employed to describe single bond ruptures in terms of intrinsic bond lifetime. Instead, a methodology that has been used to retrieve force-dependent lifetime information of the system in similar cases [44,45] was chosen, hypothesising the pulling of a stiff molecular system to fit the intrinsic lifetime of the bond τ_0_ and its energy barrier width γ.

This methodology was used on rupture event data, for the cytoskeleton anchored and the membrane tethers separately. A minimum force threshold of 20 pN and 10 pN for the cytoskeleton anchored and the membrane tethers, respectively, was set to ensure full significance of each bin in the force histograms [39].

## Results

### 1. Study sample vs. control samples

A total of 5905 force spectroscopy data were analysed for Sample 1 (HABP/HA), 687 for Sample 2 (BSA/HA), 696 for Sample 3 (untreated/HA) and 563 for Sample 4 (HABP/HAase).

The successful target rate was calculated for each sample as shown in Table 1 and was comparable in the three series of testing for each cell. In Sample 3 (untreated/HA), it was remarkably lower than in the study sample (Sample 1, HABP/HA) and the other two control samples.

**Table 1:** Successful target rate in the study and control samples. The calculated successful target rate is reported for each sample as an index of the number of successful binding events between probe and target molecule. This quantity was calculated as the percentage of curves displaying some rupture events over the total number of analysed curves.

Sample 1	Sample 2	Sample 3	Sample 4
HABP/HA	BSA/HA	untreated/HA	HABP/HAase
65.19%	67.92%	16.67%	66.11%

**Fig 4:**
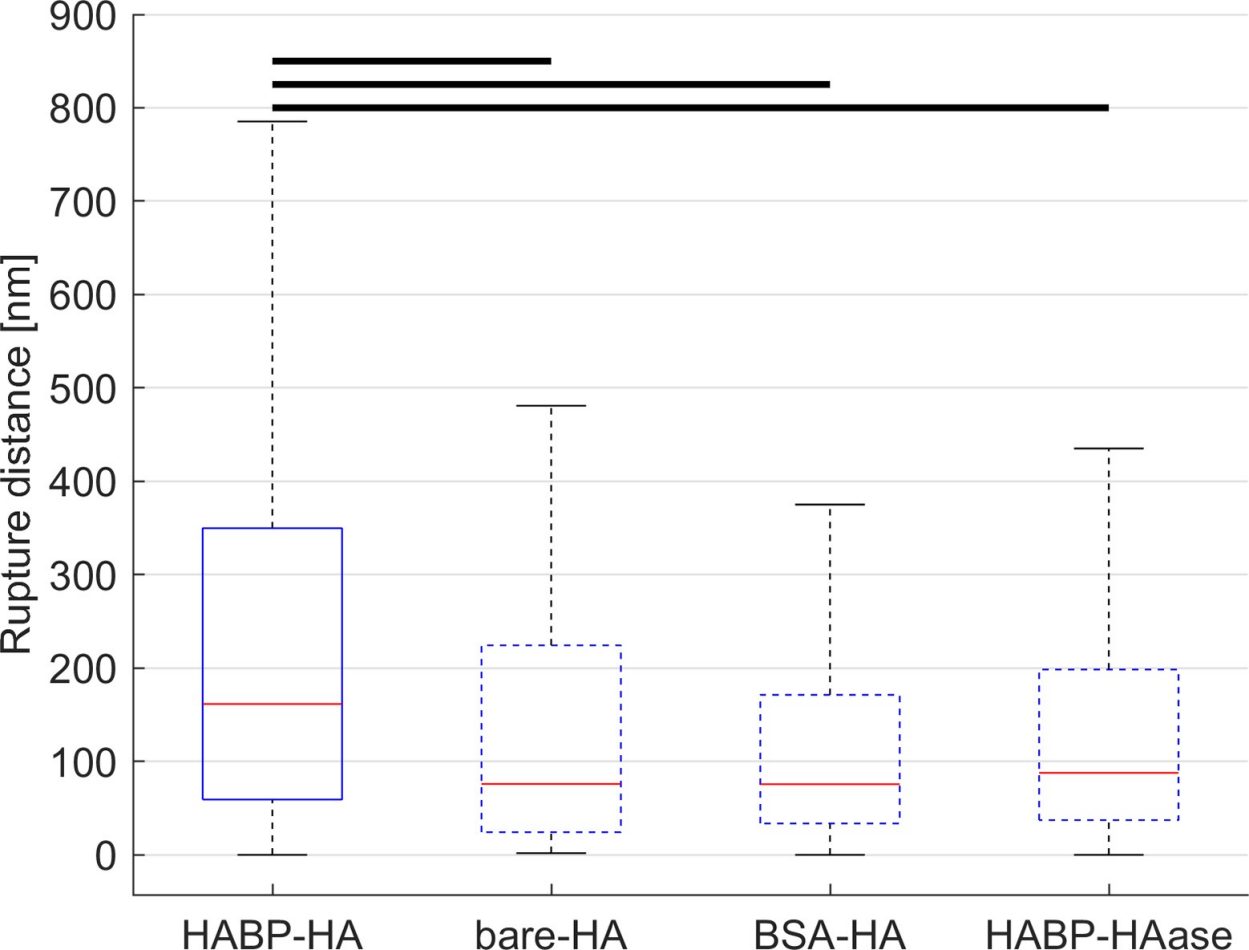
Box plot of the rupture distance from the contact point for the study and control samples. Box plot of rupture distance from the contact point for the study sample (Sample 1—HABP/HA, solid line) and the control samples (Sample 2—BSA/HA, Sample 3—untreated/HA, Sample 4—HABP/HAase, dashed line). The rupture distance from the contact point for the study sample was statistically significantly higher suggesting specificity of the bond between HABP and HA (Kruskal–Wallis test by ranks, p < .01).

The distance from the contact point at which the rupture events occurred was calculated as an indicator of the interaction specificity, with low distance values a sign of non-specific interactions. The rupture distance from the contact point for each sample was compared (Fig 4) and a statistical difference was found between the study sample and all the control samples (Kruskal–Wallis test by ranks, p < .01).

### 2. Cytoskeleton anchored vs. membrane tether ruptures

**Fig 5:**
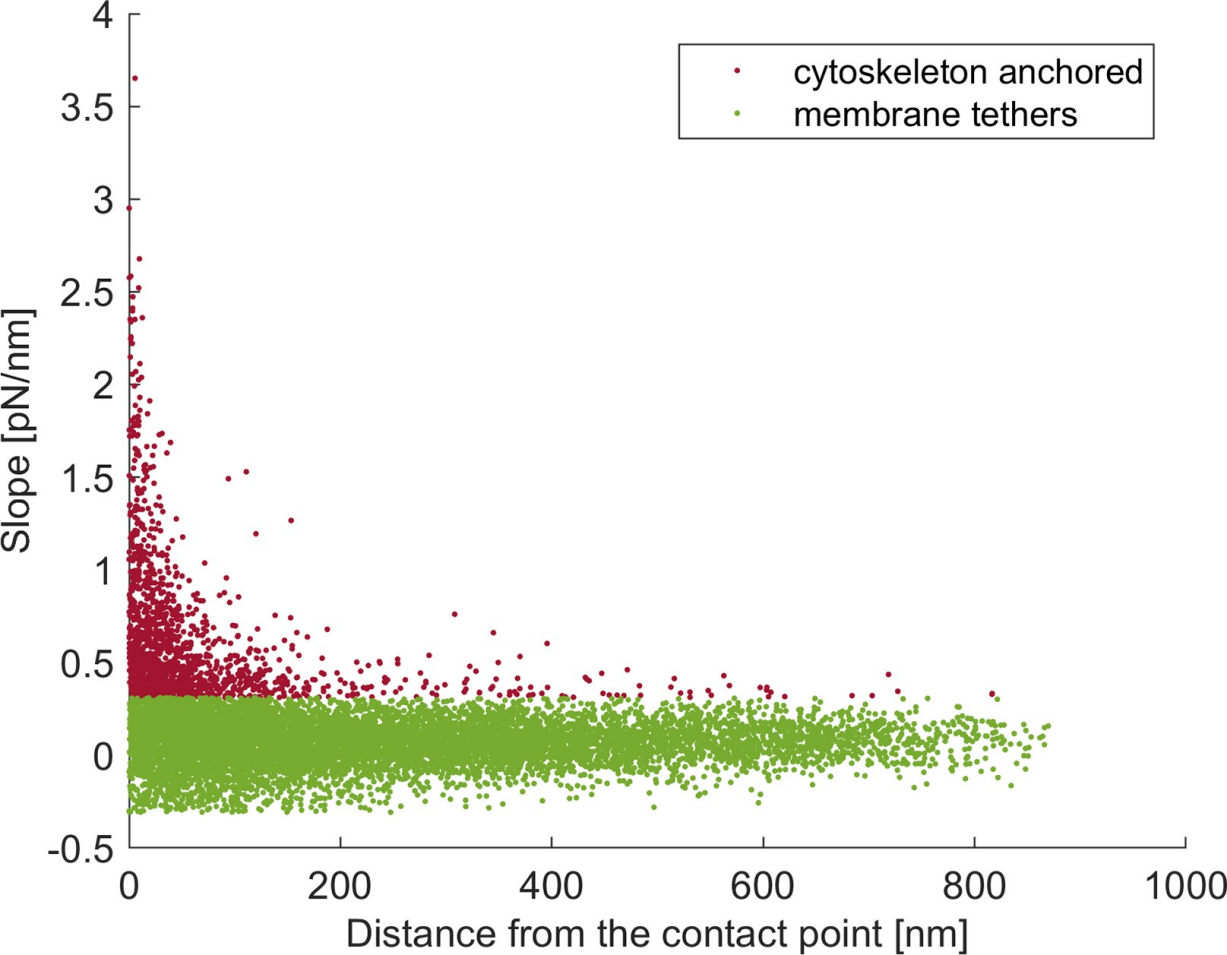
Classified rupture events in the study sample. Scatter plot of rupture events in Sample 1 (HABP/HA). The x-axis represents the distance from the contact point, the y-axis the slope of the curve before rupture. The rupture events classified as cytoskeleton anchored are shown in red, as membrane tethers in green.

In Fig 5 the rupture distance from the contact point against the slope of each rupture event of the study sample (HABP/HA) is shown. It could be noted that few rupture events with low distance from the contact point were present, which may represent non-specific binding. A threshold could be used to rule out these events from further analysis, but a suitable threshold definition to avoid removing useful data could not be defined.

Over the total number of rupture events, 14.4% were classified as cytoskeleton anchored and 85.6% as membrane tethers, underlining that about one HA molecule every seven has an active indirect link to the actin cytoskeleton. The number of cytoskeleton anchored rupture events remained constant over the three series of testing for each cell (data not shown).

**Fig 6:**
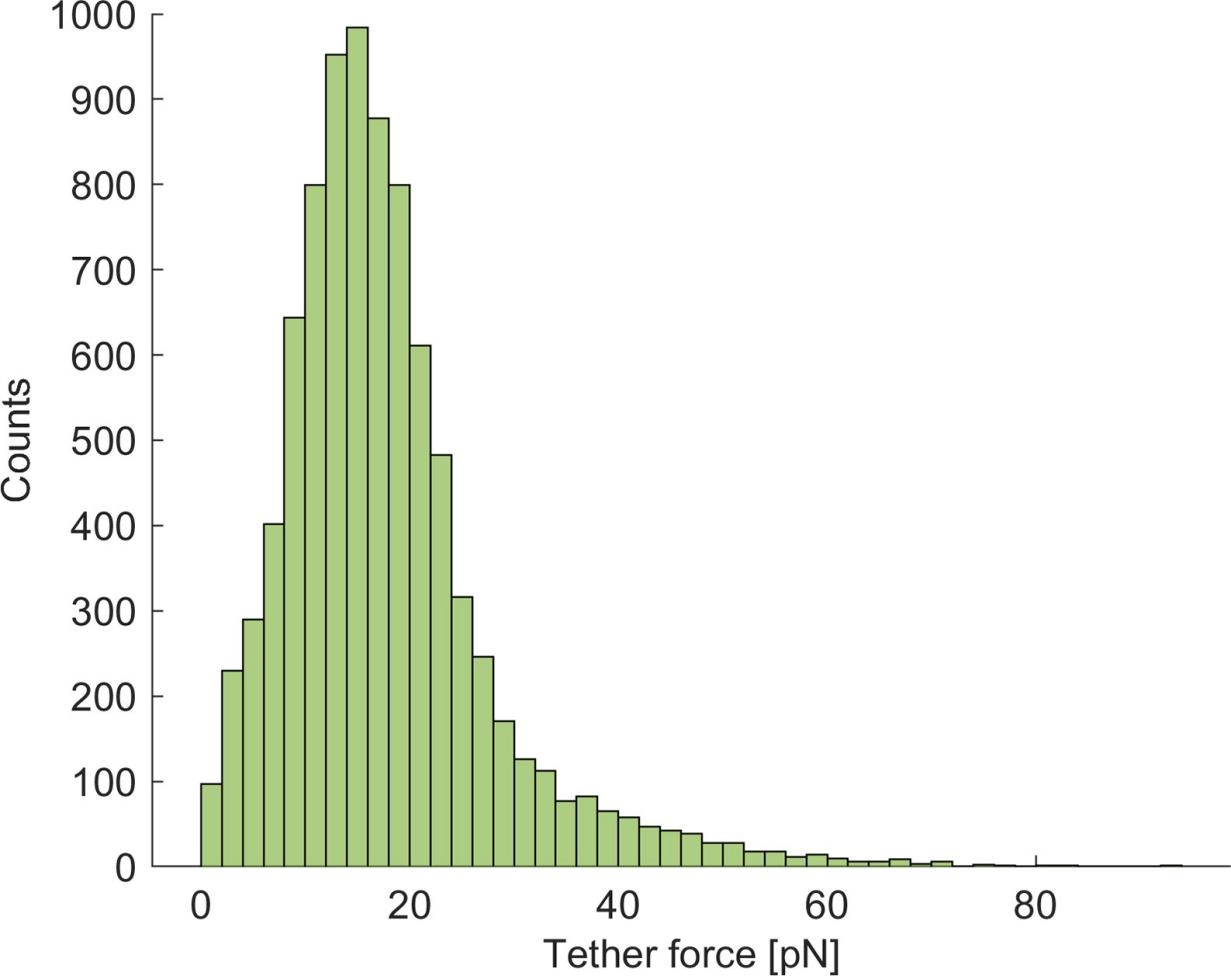
Histogram of the membrane tether force. Probability histogram of the membrane tether force in Sample 1 (HABP/HA). The median value is equal to 16 pN, the IQR to 10 pN.

Tether formation can be characterised by the tether force, i.e. the sudden release of force due to tether rupture. If multiple tethers are formed, the tether force corresponds to the force step between one tether and the subsequent one [40]. The average (median) calculated tether force was 16 pN, with a dispersion (interquartile range or IQR) of 10 pN (Fig 6). The average (median) calculated tether length was equal to 199 nm, with a spread distribution (IQR of 291 nm and maximum values up to 870 nm, data not shown).

### 3. Single and multiple rupture events

A further classification undertaken on the rupture events regarded their occurrence as single or multiple events within one retraction curve. Over the total number of successfully targeted retract curves in Sample 1 (HABP/HA), about four in every seven showed single ruptures. Two to six rupture events could be counted in about 99% of the curves in the multiple sample. 14.1% of the single rupture events were classified as cytoskeleton anchored and 85.9% as membrane tethers; 14.5% of the multiple rupture events were classified as cytoskeleton anchored and 85.5% as membrane tethers.

**Fig 7:**
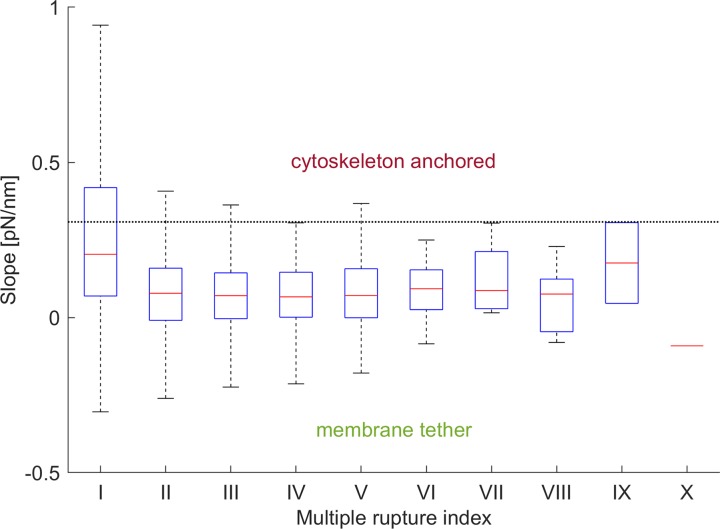
Box plot of the multiple rupture events slope. Box plot of the slope of multiple rupture events. The slope of the first (I) event is statistically significantly different from the slope of the second to sixth (II-VI) rupture events (Kruskal–Wallis test by ranks, p < .01). The size of samples VII-X was too small for accurate statistical analysis as less than 1% of data were in these samples.

The slope of the first (I) rupture event was statistically significantly higher than the slope of the second to sixth (II-VI) rupture events (Fig 7, Kruskal–Wallis test by ranks, p < .01). The size of samples VII-X was too small for accurate statistical analysis as less than 1% of multiple rupture data showed more than six rupture events. Moreover, the first rupture in cases of multiple ruptures would classify as a cytoskeleton anchored one with ~2.5-fold higher rate (36.4%) than in case of single ruptures.

### 4. Intrinsic bond characterisation

**Fig 8:**
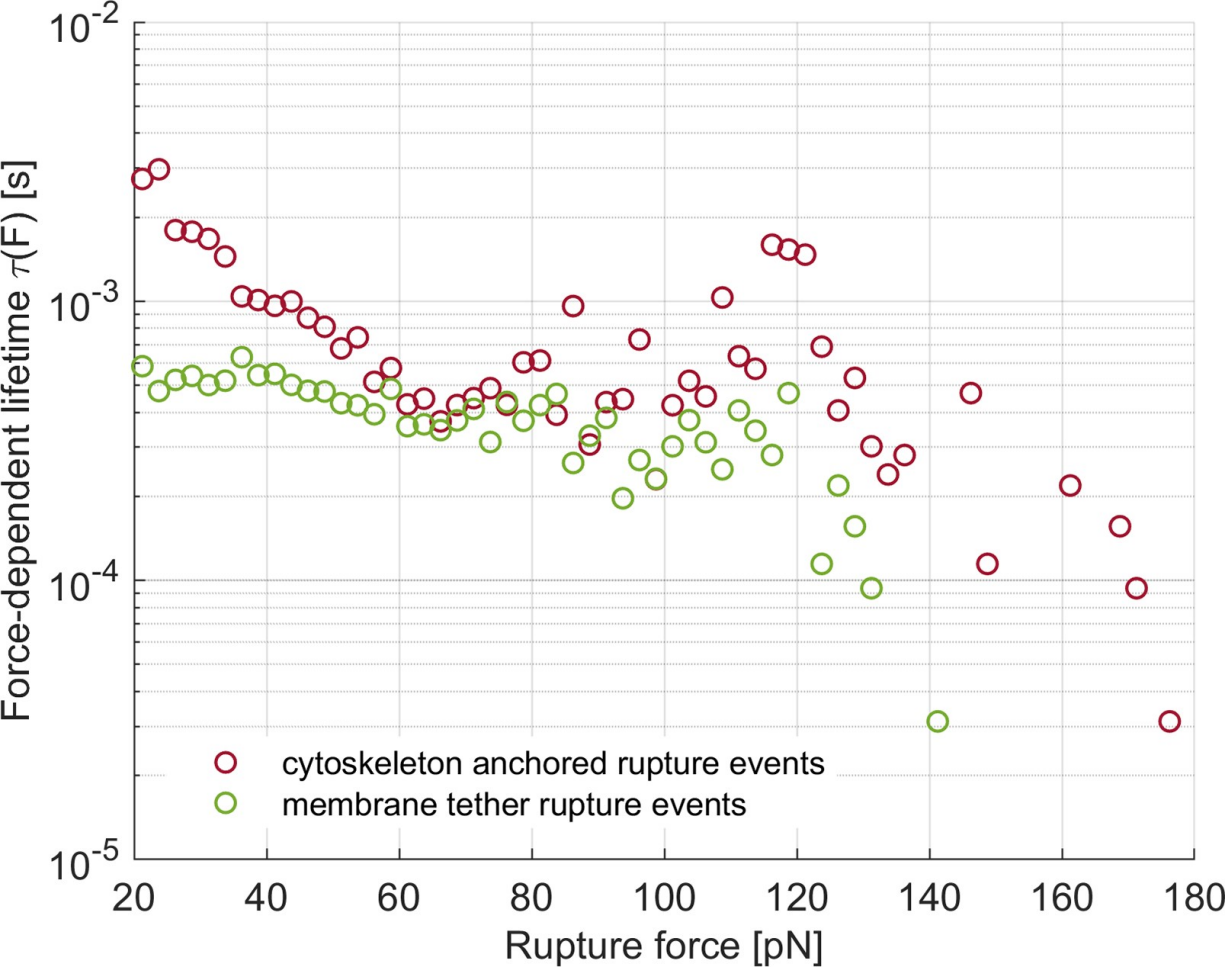
Force-dependent bond lifetime. The calculated force-dependent lifetime of the system for the cytoskeleton anchored (red circles) and the membrane tether (green circles) rupture events is shown. Calculated points did not collapse on a master curve as expected for single-exponential molecule kinetics.

The methodology proposed in [44,45] was employed to investigate the intrinsic bond lifetime τ_0_ and the energy barrier width γ. For this aim, the force-dependent system lifetime τ was calculated from the force histograms. The obtained data should collapse onto a master curve if the molecule kinetics can be explained by a single exponential function [45]. However, this was not the case for either the cytoskeleton anchored nor the membrane tether ruptures (Fig 8) and it was therefore not possible to calculate the intrinsic bond lifetime τ_0_ and the energy barrier width γ for either type of rupture event.

## Discussion

Hyaluronic Acid was targeted by AFM single-molecule force spectroscopy on live pre-osteoblast MC3T3-E1 cells, which are decorated with an HA-rich glycocalyx involved in mechanotransduction [28]. The anchoring of the HA molecule to the cytoskeleton was investigated to hypothesise possible mechanotransduction mechanisms.

### 1. Study sample vs. control samples

HA molecules were successfully targeted by using HABP-functionalised cantilever. The specificity of the HABP-HA bond was supported by the observation of the controls’ successful target rates, i.e. the number of force spectroscopy curves showing rupture events counted over the total number of data curves.

For the untreated cantilever in Sample 3 (untreated/HA) the successful target rate was markedly lower than in the other samples. This was because the cantilever was not functionalised, which resulted in fewer interactions recorded between the probe and the sample. The other control samples showed, however, successful target percentages comparable to the study sample. This could be explained by the presence of non-specific interactions caused by the cantilever functionalisation process. The distance from the contact point at which the rupture event occurred was therefore calculated to corroborate this hypothesis and resulted in statistically significantly lower values in all control samples with respect to the study sample. Cantilevers treated with BSA showed weak interactions, which could not sustain the increasing pulling force of the cantilever. If cells were treated with HAase similar weak interactions were observed, possibly linked to the cantilever interacting with membrane domains exposed by the removal of the glycocalyx. The specificity of the bond between HABP and HA in the study sample was hence considered to be satisfied.

The successful target rate was comparable in the three series of testing for each cell, suggesting that no HA fragments remained attached to the cantilever causing the saturation of binding sites. The amount of HABP absorbed on the cantilever was not measured. However, it was observed that the successful target rate would not decrease during series of testing on the same or on different cells throughout the experiments. This would suggest that the cantilever binding sites were not saturated or damaged for the duration of the experiments and that the chosen concentration of functionalisation molecule was appropriate.

### 2. Cytoskeleton anchored vs. membrane tether ruptures

It was possible to distinguish between cytoskeleton anchored and membrane tether ruptures by analysing the slope preceding the observed ruptures during retraction of the cantilever from the cell. Both categories were heterogeneous: the HA can be linked to the membrane by its synthases (HAS) or the receptor CD44 and these could anchor to the cytoskeleton with different molecules; the local composition and mechanical properties of the membrane could affect the membrane tether characteristics and rupture force. This heterogeneity was also observed in the evaluation of the single chemical bond intrinsic characteristics, where the interplay of different molecules was hypothesised.

About one HA molecule per every seven showed an active indirect link to the actin cytoskeleton, which could occur through the CD44 receptor or the HA synthases as it was not possible to distinguish between the two cases when pulling on the HA molecule. Moreover, if the linkage was through CD44, the linker molecule could differ (i.e. ERM family, merlin or oligomers of these proteins [22]) making the category of cytoskeleton anchored bonds rather heterogeneous. Future work should be directed to the use of knockdown approaches to test the relative contribution of each candidate molecule for hyaluronic acid/cytoskeleton linkage, as it would allow a more specific classification of the rupture events. The number of cytoskeleton anchored rupture events remained constant over the three series of testing for each cell, suggesting that if the HA/actin binding were severed during measurements they would reform within the time frame of 30 s corresponding to the delay between probing the same spot.

The HABP-HA bond was shown to be strong enough to pull membrane tethers. This would suggest that the HA molecule acts as a rigid linker between the cantilever and the cell membrane. This observation is in accordance with the conformation of the HA molecule, in which repeated disaccharide units form a linear structure organised as a random coil [46]. No domain unfolding or mechanical denaturation are therefore expected, as they were not observed in the retraction curves.

Membrane tethers are structures involved in cell-cell adhesion, communication and motion which originate from the cell membrane [8]. The membrane surface tension regulates various intracellular events and its maintenance is finely regulated [40,47]. The cell membrane is capable of accommodating small and large variations in the surface tension using different mechanisms: small surface tension fluctuations are buffered by use of the membrane reservoir, i.e. extra membrane stored in the form of undulations, folds, ruffles, microvilli and *caveolae*; large variations are facilitated by membrane material dynamic recycling mechanisms. The ability to create membrane tethers resides in the existence of the membrane reservoir, which is affected by the membrane composition and mechanical properties, the interactions with the cytoskeleton and the lipid bilayer turnover.

It has been shown that the tether growth once started does not depend on the chemical nature of the attachment between the force transducer and the cell membrane [8]. In previous works employing AFM, membrane tethers were pulled by cantilevers which were either untreated [8] or treated with non-specific adhesive molecules [8,40]. In the case of untreated cantilevers, the contact with the cell was maintained for 2–30 s before retracting to facilitate the tether formation. In the present study, no prolonged contact was performed and the HABP/HA binding behaved similarly to non-specific adhesion. Therefore it is hypothesised that the HABP/HA binding is strong enough to allow for the initiation of tethers, despite pulling through the HA molecule and not directly on the membrane.

The average tether force calculated in this study was 16 pN, falling in the range of 10–60 pN measured in the literature for various cells [8]. The quantity dispersion was hypothesised to represent the membrane heterogeneity, both in terms of composition and mechanical properties. The average tether length for osteoblasts differentiated from human mesenchymal stem cells was measured as 4.0 ± 1.1 μm by laser optical tweezers [47]. The average tether length calculated in this study was however much shorter, equal to 199 nm, with a much-dispersed distribution. This is probably due to differences in the employed protocols [47], both in terms of chemical attachment of the probe to the membrane and in terms of translational movement of the probe in respect to the cell. In fact, in the present protocol the cantilever was kept close to the cell surface and was translated between subsequent spots of the testing grid. For this reason, the tethers were pulled vertically for a limited range (~1 μm) and not allowed to develop in full length.

Three scenarios could, therefore, occur: i) all the tethers would rupture during the vertical movement; ii) some of the tethers would be maintained during the lateral cantilever movement and would be ruptured during the translation; iii) some of the tethers would be maintained during the lateral cantilever movement and would still be present during indentation of the subsequent test grid spot. The first two cases were difficult to discern due to the hydrodynamic effect: if some residual tethers were still be bound to the cantilever, the zero force condition (i.e. baseline) would not have been reached on the retract curve, similar to previous observations [8]. Similar outcomes would, however, be observed in the case of the hydrodynamic effect, as the cantilever would be pushed to bend up due to the liquid resistance when moving downwards and to bend down when moving upwards. In the present protocol, it was chosen to correct the retract curves for the hydrodynamic effect, having observed that the offset between baselines was comparable to previous indentation experiments with untreated cantilevers. Therefore, longer tethers in the second scenario were overlooked and the total average tether length probably underestimated. Data belonging to the third scenario were excluded from the analysis, as recognisable from unexpected peaks during the cantilever extend/cell indentation region.

### 3. Single and multiple rupture events

It was possible to hypothesise the presence of patterns in the HA molecule pulling by analysing the multiple rupture events. Multiple rupture events were observed in about 60% of the force spectroscopy curves, with most curves showing from two to six rupture events. The slope of the first rupture event was statistically significantly higher than the slope of the following rupture events and showed a higher incidence of cytoskeleton anchored ruptures than in the case of single rupture events. These results would suggest that a cytoskeletal bond needs to be broken to improve the ability to pull multiple tethers from the cell membrane. Similar results were obtained on red blood cells and it was hypothesised that at the beginning of tether formation the link with the cytoskeleton would be partially broken and further tether elongation would be accompanied by the elongation of the intact components of the cytoskeleton [24]. The resulting tether would therefore not be completely disconnected from the cytoskeleton and possibly maintain an actin cytoskeleton core, analogous to what was observed in the formation of HA-rich microvilli [5–7].

Different mechanotransduction mechanisms involving HA could be hypothesised from the observed results. Single cytoskeleton anchored rupture events represent HA molecules linked to the cytoskeleton through transmembrane components and therefore transmitting mechanical stimuli into the inner cell compartments, with "decentralised" mechanosensing at sites distinct from the cell surface [16,19]. Single membrane tethers would conversely be examples of "centralised" mechanosensing, with the glycocalyx molecules directly connected to areas of the membrane where an abundance of signalling molecules reside [16]. Finally, multiple rupture events with an initial link to the cytoskeleton might represent microvilli-like structures with an actin-rich core, similar to the ones observed for cells over-expressing HA synthases and hypothesised to have a signalling role [2,5–7].

Future work might be directed towards experimental testing of these hypotheses. Recently developed advanced imaging techniques have been used for subcellular processes visualisation. Fluorescence-resonance energy transfer (FRET) technology, for example, was used in the context of mechanotransduction to analyse relevant signalling molecules, such as calcium signalling and pathways associated with cytoskeleton remodelling. Moreover, FRET-based force sensors able to detect intracellular tension were also designed relying on the unfolding of proteins at strategic cell locations such as focal adhesions or adherens junctions [48].

### 4. Intrinsic bond characterisation

It was not possible to calculate the intrinsic bond lifetime τ_0_ and the energy barrier width γ for either type of rupture event, as the molecular kinetics could not be explained by a single exponential function [45]. This result would suggest that both categories were heterogeneous and contained events of different nature. As previously discussed, the cytoskeleton anchor link might be one or more proteins of the ERM family or merlin and this could result in different bond characteristics, while the membrane tethers may or may not conserve an actin-rich core. Moreover, non-specific interactions might have played a role, as they were not ruled out before the analysis.

## Conclusions

In this study, HA molecules on the surface of pre-osteoblast cells were investigated by single molecule force spectroscopy. HA molecules were successfully targeted by using HABP-functionalised cantilevers. Two types of rupture events were observed when the HA molecules were anchored to the cytoskeleton or to the membrane. Mechanotransductive mechanisms were hypothesised depending on the observed rupture patterns.

The set of experiments presented in the present manuscript focused on the evaluation of the mechanical link between the HA and the cytoskeleton and represented a preliminary stage to further investigate this attachment from a biochemical point of view. The present findings support the idea of the HA having a structural role and being able to organise and support the cell membrane as an external cytoskeleton deeply involved in the glycocalyx-mediated mechanotransduction.
